# Partizipative Gesundheitsforschung: Ursprünge und heutiger Stand

**DOI:** 10.1007/s00103-020-03264-y

**Published:** 2020-12-17

**Authors:** Michael T. Wright

**Affiliations:** Katholische Hochschule für Sozialwesen Berlin (KHSB), Köpenicker Allee 39–57, 10318 Berlin, Deutschland

**Keywords:** Forschungsmethoden, Überblicksartikel, International Collaboration for Participatory Health Research, Partizipation, Gesundheitliche Chancengleichheit, Research methods, Review articles, International Collaboration for Participatory Health Research, Participation, Health equity

## Abstract

Partizipative Gesundheitsforschung (PGF) wird international – und seit einigen Jahren auch in Deutschland – zunehmend als Möglichkeit wahrgenommen, wissenschaftliche Erkenntnisse zu erzeugen, die unmittelbar zur Verbesserung von Gesundheitschancen für sozial benachteiligte Bevölkerungsgruppen beitragen. Zentrales Merkmal der PGF ist die direkte Beteiligung der Menschen am Forschungsprozess, deren Arbeits- oder Lebensverhältnisse Gegenstand der Forschung sind. Dieser Beitrag gibt einen Überblick über Ursprünge, Grundlagen und aktuelle Entwicklungen der PGF. Wissenschaftliche Literatur aus der internationalen Diskussion um die PGF wird im Sinne eines narrativen Reviews rezipiert, der Schwerpunkt liegt auf Überblicksarbeiten und Publikationen der International Collaboration for Participatory Health Research.

Das Review ergibt, dass eine wachsende Anzahl von Forschenden im Gesundheitsbereich partizipativ arbeitet. Es ist auch deutlich erkennbar, dass ein eigenständiger wissenschaftlicher Diskurs und diverse Vereinigungen partizipativ Forschender sich in vielen Ländern etabliert haben. Folgende aktuelle internationale Entwicklungen sind hervorzuheben: Konsolidierung und Vernetzung, Klärung der Frage, was Partizipation in der Forschung bedeutet, Evaluation der Auswirkungen und des Mehrwerts der PGF, Weiterentwicklung der PGF in spezifischen Anwendungsbereichen und die Klärung ethischer Fragen in der PGF.

## Abstract (en)

Participatory health research (PHR) is receiving increasing attention internationally as an approach for generating scientific knowledge to promote health equity for marginalized groups. A similar trend has been observed in Germany over the past several years. The primary characteristic of PHR is the direct participation of those people in the research process whose work or living conditions are the subject of the research. This article provides an overview of the origins, foundational principles, and current developments in the field of PHR. The international research literature is discussed in the form of a narrative review with a focus on previous review articles and publications from the International Collaboration for Participatory Health Research.

The review indicates that a growing number of health researchers are engaged in participatory research and that a scientific discourse and various organizations have developed in many countries focused specifically on PHR. The following trends are of particular importance: consolidation and networking; clarifying the question of what constitutes participation in research; evaluating the impact and added value of PHR; adapting PHR to specific areas of application; and clarifying ethical questions in PHR.

## Einführung

Internationale Forschungsergebnisse zeigen eindeutig, dass Gesundheitschancen nach einem sozialen Gradienten verteilt sind. Das heißt, Menschen mit einem höheren Sozialstatus (z. B. aufgrund von Bildung und Einkommen) leben länger und werden seltener krank als Menschen mit einem niedrigeren Status. Diesen Zustand zu verstehen und zu verändern, ist zu einem zentralen Gegenstand der gesundheitswissenschaftlichen Forschung und Praxis geworden [1–3]. Vor allem die Gesundheitsförderung hat sich zum Ziel gesetzt, die Gesundheit der Bevölkerung durch Veränderung der sozialen Umstände zu verbessern. Der Schwerpunkt liegt auf den Lebensbedingungen marginalisierter Bevölkerungsgruppen. Dabei ist es notwendig, komplexe Prozesse der Ausgrenzung auf kollektiver Ebene (Stadtteil, Kommune, Gesellschaft) zu verändern, die sich beispielsweise in Form eines beschränkten Zugangs zu Leistungen des Gesundheitswesens zeigen [4–6].

Partizipative Gesundheitsforschung (PGF) wird international – und seit einigen Jahren auch in Deutschland – zunehmend als Möglichkeit wahrgenommen, wissenschaftliche Erkenntnisse zu erzeugen, die unmittelbar zur Verbesserung von Gesundheitschancen für sozial benachteiligte Bevölkerungsgruppen beitragen. Zentrales Merkmal der PGF ist die direkte Beteiligung der Menschen am Forschungsprozess, deren Arbeits- oder Lebensverhältnisse Gegenstand der Forschung sind. Damit ist nicht gemeint, die Menschen als Proband*innen oder Studienteilenehmer*innen in eine Forschung einzubeziehen, sondern als Forschungspartner*innen auf Augenhöhe. Alle Forschungspartner*innen bestimmen gemeinsam die Kernelemente des Forschungsprojekts, von der Auswahl des Forschungsschwerpunkts bis hin zur Methodenauswahl, Datenerhebung und Interpretation der Ergebnisse. Erfahrungen aus dem In- und Ausland zeigen, dass Partizipation in diesem Sinne voraussetzungsreich und deshalb oft schwer zu realisieren ist. Die Partizipation bietet jedoch eine unvergleichbare Chance, durch Forschungsprojekte Veränderungen in Gang zu setzen, sowohl bei den beteiligten Personen als auch bei den Einrichtungen und Netzwerken, zu denen sie gehören [7].

Partizipative Ansätze in der Forschung haben in verschiedenen Disziplinen Anerkennung erreicht, z. B. in den Erziehungswissenschaften, in der Organisationsentwicklung, in der Gemeinwesen- und Entwicklungsarbeit und in der Sozialarbeitswissenschaft. Partizipation in der Gesundheitsforschung wird erst seit den 1980er-Jahren zunehmend thematisiert. Dies ist auf die biomedizinische Forschung zurückzuführen, die bei Weitem die Mehrheit der Gesundheitsforschungsprojekte ausmacht. Die biomedizinische Forschung folgt einem positivistischen Paradigma, nach dem quantitativen experimentellen Untersuchungen die stärkste Aussagekraft zugemessen wird. Dieses Wissenschaftsverständnis wurde auch auf andere, nichtklinische Bereiche der Gesundheitsforschung übertragen, obwohl die Grenzen solcher Vorgehensweisen in Bezug auf die sozialen Determinanten von Gesundheit (z. B. in der Versorgungsforschung oder der Gesundheitsförderungsforschung) verschiedentlich aufgezeigt wurden [8–10]. In der Folge genießen qualitative und partizipative Forschungsansätze der Gesundheitsforschung bisher nicht die gleiche Anerkennung und gelten daher häufig nicht als zuverlässige Quellen der Evidenz [11, 12].

In diesem Beitrag wird der aktuelle Stand der PGF beschrieben. Nach einer Erklärung der Methodik werden Ursprünge und Grundlagen der PGF erklärt und dann aktuelle internationale Entwicklungen vorgestellt. Der Beitrag schließt mit einem Fazit.

## Methodik

Das Ziel dieses Beitrags ist in Form eines narrativen Literaturreviews eine Übersicht über Ursprünge, Grundlagen und aktuelle Entwicklungen in der PGF aus internationaler Perspektive zu präsentieren. Wie bei allen Reviews dieser Art sind die Auswahl und die Auslegung der Literatur von der Expertise und Erfahrung des Autors abhängig. In diesem Fall ist der Autor Mitbegründer des Netzwerks Partizipative Gesundheitsforschung (PartNet) und der International Collaboration for Participatory Health Research (ICPHR). Er leitete über mehrere Jahre die Geschäftsstellen beider Einrichtungen und ist aktuell für die Geschäftsstelle der ICPHR zuständig. Ein Schwerpunkt seiner wissenschaftlichen Arbeit ist die Übertragung international anerkannter Ansätze der partizipativen Forschung auf deutsche Wissenschaft und Praxis. Der Autor hat durch die Schwerpunktlegung auf Übersichtsarbeiten und auf Publikationen der ICPHR versucht, Erkenntnisse und Positionen darzustellen, die aktuell für einen breiten Konsens unter Fachleuten stehen.

## Ursprünge und Grundlagen

Die PGF entstand aus partizipativen Ansätzen der Sozialforschung, deren europäische und nordamerikanische Wurzeln in der Aktionsforschung, auch Handlungsforschung genannt, liegen. Einer der prominentesten und bis in die heutige Zeit einflussreichsten Vertreter ist Kurt Lewin (1890–1947), gebürtiger Berliner aus jüdischer Familie. Er erarbeitete die Basis für die Verzahnung von Forschung und Handlung mit dem Ziel, vor allem die Umsetzung demokratischer Werte in der Gesellschaft und die Optimierung von Organisationsabläufen und -strukturen zu fördern. Im Sinne der Aktionsforschung bedeutet „Aktion“ oder „Handlung“ jene Versuche, soziale Probleme zu lösen oder zu lindern. Aktionsforschung verfolgt explizit das Ziel, neue Erkenntnisse über soziale Probleme zu gewinnen, um Handlungsoptionen zu konzipieren. Oft ist die Umsetzung dieser Optionen (auch) Gegenstand eines Forschungsprojekts. Die Arbeit von Lewin wurde zum großen Teil in englischer Sprache verfasst, da er aufgrund der Verfolgung durch Nationalsozialisten in die USA auswandern musste, wo er seine Arbeit fortsetzte. In seiner neuen Heimat prägte er den Begriff *Action Research*. Die Aktionsforschung erreicht unter dem Einfluss der Kritischen Theorie^1^ (vor allem der Kritischen Psychologie) und der Studentenbewegung Ende der 1960er- bis in die 1970er-Jahre ihren Höhepunkt in Deutschland. Sie verlor jedoch an Bedeutung, als das politische Klima konservativer wurde und der Rechtfertigungsdruck seitens der herkömmlichen Wissenschaft in den 1980er-Jahren zunahm. Bis in die 2000er-Jahre waren nur wenige Aktionsforscher*innen noch aktiv [13, 14].

Während in Deutschland die Aktionsforschung kaum noch eine Rolle spielte, entwickelten sich im Ausland Formen von Wissenschaft weiter, die – unter dem Einfluss von Menschen außerhalb wissenschaftlicher Einrichtungen – die Entwicklung von Handlungsoptionen zur Linderung oder Lösung sozialer Probleme in den Mittelpunkt stellten. Wichtige Forschungsansätze (und deren prominente Vertreter*innen) sind: Participatory Rural Appraisal (R. Chambers), emanzipatorische Forschungsansätze (P. Freire, O. Fals Borda), Aktionsforschung in der Organisationsentwicklung (K. Lewin, H. Bradbury), Aktionsforschung in der Pädagogik (S. Kemmis, J. McNiff), Human Inquiry und Cooperative Inquiry (P. Reason), Appreciative Inquiry (D. Cooperrider), Community-based Participatory Research (N. Wallerstein, M. Minkler, B. Israel), Action Science (C. Argyris, D. Schön), konstruktivistische Forschung (Y. Lincoln), feministische Forschung (P. Lather), Empowerment Evaluation (D. Fetterman) und Democratic Dialogue (B. Gustavsen). Vertreter*innen der PGF gründen ihre Arbeit auf einer oder mehreren dieser Traditionen. Es ist üblich, dass sie je nach Kontext Methoden und Konzepte aus verschiedenen Traditionen einsetzen, um ihre Arbeit optimal an den spezifischen Forschungszusammenhang anpassen zu können. Allen Ansätzen ist eine Nähe zu sozialen Bewegungen gemein, die sich für eine demokratische und inklusive Gesellschaft einsetzen. Sie teilen auch die kritische Haltung, dass Wissenschaft sich nicht in der Einhaltung bestimmter epistemologischer oder methodologischer Kriterien erschöpft, sondern als Mittel zur Verbesserung der Lebensbedingungen von Menschen durch neue Erkenntnisse dient. Zudem verbinden folgende zentrale Merkmale der Forschungspraxis die verschiedenen Ansätze:

1. Der Erkenntnisgewinn wird unmittelbar mit der Entwicklung und Erprobung neuer Handlungsmöglichkeiten verknüpft, um die Arbeitsweisen oder Lebensumstände der Beteiligten zu verbessern.
2. Alle Beteiligten arbeiten auf Augenhöhe, um möglichst alle Phasen eines Forschungsprozesses gemeinsam zu konzipieren und durchzuführen. In diesem Sinne ist die Forschungsarbeit partizipativ. Alle Beteiligten werden als Forscher*innen bezeichnet. Die unterschiedlichen Hintergründe der Forscher*innen können durch genauere Bezeichnungen wie akademisch Forschende (Wissenschaftler*innen), Peer-Forschende oder Lebenswelt-Expert*innen etc. verdeutlicht werden.

Oft wird gefragt, wie partizipativ ein Forschungsvorhaben sein muss, um als PGF-Projekt gelten zu können. Um diese Frage zu beantworten, sind mehrere „Stufenmodelle“ entwickelt worden, die partizipativ Forschende bei der Reflexion des Grades der erreichten Partizipation unterstützen. Grundsätzlich gilt, dass die Einflussnahme der Menschen, deren Arbeits- oder Lebensumstände im Mittelpunkt der Forschungsarbeit stehen, auf alle Phasen des Forschungsprozesses maximiert wird. In der Praxis sind die Partizipationsmöglichkeiten jedoch aus diversen Gründen eingeschränkt. Internationale Erfahrungen zeigen, dass Partizipation oft behauptet wird, doch lediglich eine Scheinpartizipation vorhanden ist. Eine kritische Reflexion der Forschenden über das eigene Handeln auf der Grundlage der Stufenmodelle soll eine Scheinpartizipation verhindern [15–18].

**Figure 1:**
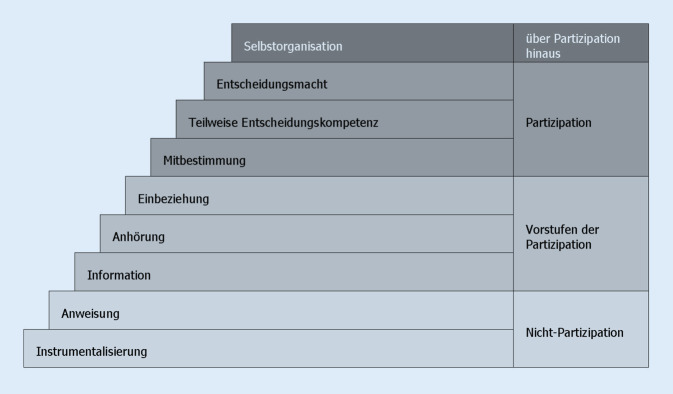

In deutscher Sprache liegt ein neunstufiges Modell vor, das unter Berücksichtigung gängiger internationaler Modelle und auf der Basis empirischer Arbeiten aus Deutschland entwickelt wurde ([19]; Abb. 1).

Das Modell besteht aus 9 Stufen, die in 4 Bereiche eingeteilt sind: Nichtpartizipation, Vorstufen der Partizipation, Partizipation und über Partizipation hinaus. Die ersten 2 Stufen sind der Nichtpartizipation zugeordnet. Hier haben die Menschen, um die es in der Forschung geht, gar keinen Einfluss auf den Forschungsprozess. Bei Stufe 1 (Instrumentalisierung) werden Menschen ohne Rücksicht auf mögliche negative Konsequenzen an der Forschung beteiligt. Bei Stufe 2 (Anweisung) sind die beforschten Menschen dazu verpflichtet, entsprechend den Erwartungen der (akademisch) Forschenden zu handeln. Aus forschungsethischen Gründen verbieten sich diese ersten beiden Stufen – auch in der nichtpartizipativen Forschung.

Forschungsethische Standards werden bei den Vorstufen der Partizipation (Stufen 3–5) eingehalten und die Perspektiven der beforschten Menschen werden zunehmend wahrgenommen. Auf Stufe 3 (Information) werden die Beteiligten über das Forschungsvorhaben ausführlich und in einer verständlichen Sprache informiert; damit werden die Bedingungen für eine informierte Einwilligung erfüllt. Dieser Informationstand ist die Voraussetzung für eine stärkere Beteiligung der Beforschten durch Anhörung (Stufe 4) und Einbeziehung (Stufe 5). Eine Anhörung bedeutet beispielsweise, dass Gespräche mit einer Selbsthilfegruppe oder anderen Vertretungen der beforschten Menschen stattfinden. Dabei werden Forschungsfragen erörtert oder Interventionskonzepte besprochen, die erforscht werden sollen. Solche Gespräche dienen u. a. der Spezifizierung und Klärung des Forschungsdesigns. Bei einer Einbeziehung ist der Austausch intensiver, beispielsweise durch einen formaleren Rahmen für Diskussionen oder wiederholte Gesprächsrunden.

Erst auf der Ebene der Partizipation (Stufen 6–8) haben die Menschen, die beforscht werden, einen formalen, unmittelbaren Einfluss auf das Forschungsprojekt. Bei der Mitbestimmung (Stufe 6) kann beispielsweise eine Forschungskooperation zwischen einer wissenschaftlichen Einrichtung und Selbsthilfegruppe zustande kommen. Die teilweise Übertragung der Entscheidungsmacht (Stufe 7) wird z. B. dadurch umgesetzt, dass einige Bestandteile der Forschung unter der Leitung der beforschten Menschen realisiert werden, wie Teile der Datenerhebung oder Datenauswertung, die Rekrutierungsstrategie oder Formen der Ergebnisverwertung. Bei der Entscheidungsmacht (Stufe 8) bestimmen Menschen aus der Gruppe der Beforschten alle wesentlichen Bestandteile des Forschungsprojekts mit, z. B. indem Vertreter*innen der Gruppe gleichberechtigte Mitglieder des Forschungsteams sind.

Bei der Selbstorganisation (Stufe 9) handelt es sich um Studien, die von den beforschten Menschen eigenständig konzipiert und durchgeführt werden. Diese Form von Forschung wird als *betroffenenkontrollierte Forschung* bezeichnet.

Das Stufenmodell ist keine Rating-Skala, die von Externen angewendet wird, um den Grad der Partizipation eines Projekts von außen zu beurteilen. Vielmehr ist das Modell als Instrument der Selbstreflexion konzipiert, das im Laufe eines partizipativ ausgerichteten Forschungsprojekts eingesetzt werden kann, um die Qualität der Zusammenarbeit in Bezug auf die relative Einflussnahme der verschiedenen Beteiligten kritisch zu reflektieren. Wichtig dabei ist, dass nicht ein Teil der Partner*innen (z. B. die beteiligten Wissenschaftler*innen) alleine die Stufe der erreichten Partizipation der anderen einschätzen, sondern dass alle Beteiligten in ihrer jeweiligen Rolle die Möglichkeit haben, selbst zu beurteilen, wie ausgeprägt ihre eigene Partizipation und die Partizipation der anderen ist. Das Stufenmodell kann auch bei der Planung eines Forschungsvorhabens genutzt werden, um die beabsichtigte Partizipation der verschiedenen Partner*innen darzustellen. Nicht alle Beteiligten wollen oder können an allen Phasen eines partizipativen Forschungsprojekts gleich beteiligt sein. In der Regel übernehmen die Partner*innen unterschiedliche Aufgaben, und der Grad der Partizipation kann über die Projektlaufzeit variieren [20].

Abhängig von Forschungsgegenstand und Zusammensetzung der Beteiligten sind 2 Schwerpunkte in der PGF erkennbar [19]:

- *Gemeinschaftsforschung *(Community-based Research): Im Mittelpunkt stehen (sozial benachteiligte) Menschen, die von Angeboten des Sozial- und Gesundheitswesens profitieren sollen. Ziel ist es, diese Menschen zu unterstützen, ihre eigene Lebenslage zu erforschen und dabei Handlungsmöglichkeiten zu entwickeln, die ihre Lage positiv verändern (oft in Zusammenarbeit mit Praxiseinrichtungen).
- *Praxisforschung *(Practitioner Research): Praktiker*innen konzipieren und setzen eigene Forschungsprojekte um mit dem Ziel, die eigene Praxis zu verbessern. Dies geschieht mit oder ohne Unterstützung wissenschaftlicher Einrichtungen.

In der Regel umfassen PGF-Projekte sowohl Gemeinschafts- als auch Praxisforschung: Menschen, die von bestimmten Gesundheitsproblemen betroffen sind, arbeiten im Rahmen von Forschungsprojekten mit Praxiseinrichtungen zusammen. Ziel ist es, sowohl die Leistungen des formalen Hilfesystems als auch die Arbeits- oder Lebensverhältnisse der betreffenden Menschen zu verbessern.

## Aktuelle Entwicklungen

Eine wachsende Anzahl von Forschenden im Gesundheitsbereich arbeitet partizipativ. Ein eigenständiger wissenschaftlicher Diskurs und diverse Vereinigungen partizipativ Forschender haben sich etabliert. Dabei sind einige aktuelle internationale Entwicklungen hervorzuheben:

### Konsolidierung und Vernetzung.

Wie vor allem an der Arbeit der International Collaboration for Participatory Health Research (ICPHR) erkennbar ist, befindet sich die PGF in einer Phase der Konsolidierung und der internationalen Vernetzung. Die ICPHR wurde 2009 gegründet [22], um partizipativ Forschende aus allen Ländern zu vernetzen, um u. a. Fragen der Definition, der Evaluation, der Qualität und der Forschungsethik gemeinsam zu beantworten. Die ICPHR prägte den Begriff partizipative Gesundheitsforschung, der als Dachbegriff für die verschiedenen Ausprägungen der Partizipation in der Gesundheitsforschung fungiert. In ihren Veröffentlichungen zeigt die ICPHR die Ähnlichkeiten und Unterschiede zwischen diesen verschiedenen Ausprägungen auf [23]. Das Netzwerk Partizipative Gesundheitsforschung (PartNet) ist eine Partnerorganisation der ICPHR im deutschsprachigen Raum, die seit 2007 die gleichen Ziele verfolgt. Es sind mittlerweile auch Netzwerke in Nordamerika, in Großbritannien, in den spanischsprachigen und in den portugiesischsprachigen Ländern entstanden, die z. T. aus der ICPHR hervorgingen und auch mit ihr kooperieren.

### Klärung der Frage, was Partizipation in der Forschung bedeutet.

Angesichts der wachsenden Anerkennung der Vorteile von Partizipation in der Gesundheitsforschung bezeichnen immer mehr Forschungsprojekte ihre Arbeit als „partizipativ“, auch wenn Partizipation im Sinne von Einflussnahme auf den Forschungsprozess kaum oder gar nicht vorhanden ist [24]. Häufig wird der Einsatz von qualitativen Methoden (wie Leitfadeninterviews oder Gruppendiskussionen) als Ausdruck eines partizipativen Vorgehens genannt. Qualitative Forschung ist jedoch nicht partizipativer als quantitative Forschung, auch wenn sie subjektive und kontextspezifische Aspekte eines Themas besser beleuchten kann. Neuere Arbeiten befassen sich mit der Festlegung von Kriterien für die Beurteilung des Vorhandenseins von Partizipation und der Qualität des partizipativen Prozesses, um mehr Klarheit in die Forschungspraxis zu bringen [25–27].

### Evaluation der Auswirkungen und des Mehrwerts der PGF.

Die Durchführung von PGF-Projekten wird oft damit begründet, dass PGF im Vergleich zur üblichen Forschung mehr Ansatzpunkte für Veränderungsprozesse offenlegt. Inwiefern diese Begründung korrekt ist und wie PGF-Projekte evaluiert werden können, um ihre Auswirkungen (Research Impact) zu erfassen, ist ein zentrales Thema internationaler Bestrebungen. Es liegen diverse Modelle für die Konzeptualisierung und Erfassung der spezifischen Auswirkungen der PGF vor [28, 29]. PartKommPlus – Forschungsverbund für gesunde Kommunen – das bislang größte Forschungsvorhaben der PGF in Deutschland – hat diese Modelle in verschiedenen Teilprojekten in die Praxis umgesetzt und an deutsche Forschungskontexte angepasst.

### Weiterentwicklung der PGF in spezifischen Anwendungsbereichen.

Partizipation in der Forschung muss je nach Anwendungsbereich und Gruppe von den beteiligten Menschen unterschiedlich realisiert werden. Um spezifische Anforderungen und Umsetzungsmöglichkeiten zu klären, sind mehrere Veröffentlichungen und internationale Kooperationen entstanden – beispielsweise Kids in Action, eine Vereinigung von über 50 Projekten aus verschiedenen Ländern, die PGF mit Kindern beschreibt und konzeptuell und methodisch fundiert [30]. Partizipation in der epidemiologischen Forschung entwickelt sich ebenfalls zu einem eigenständigen Anwendungsgebiet, das die Bedeutung von Mixed Methods hervorhebt, z. B. in der Gesundheitsberichterstattung [31, 32]. Mit der Nähe zur Praxis wird die PGF auch im Bereich der Implementationsforschung (Implementation Research, Translational Research) zunehmend wichtig [33]. Ein weiterer Schwerpunkt der PGF speziell in Organisationen des Gesundheitswesens liegt auf Themen der Versorgungsforschung. Dabei sind spezifische Formen der Partnerschaft und Methodik zu berücksichtigen [34].

### Klärung von ethischen Fragen in der PGF.

Partizipation in der Gesundheitsforschung wirft besondere forschungsethische Fragen auf. Wenn auch Menschen außerhalb wissenschaftlicher Einrichtungen als Mitglieder des Forschungsteams mitarbeiten, gehört das Forschungsprojekt auch ihnen. Wo und wie die Ergebnisse des Forschungsprojekts veröffentlicht werden, ist dann Aushandlungssache im Team und nicht nur eine Entscheidung der beteiligten Wissenschaftler*innen. Die Vergütung der Mitarbeit ist ein ebenso wichtiges Thema: Wenn nur Hochschulmitarbeiter*innen bezahlt werden, stellen sich Fragen der Gerechtigkeit und der Würdigung der geleisteten Forschungsbeiträge. Peer-Forschende sind durch ihre Doppelrolle als Forschende und Mitglieder der beforschten Gruppe mit spezifischen ethischen Herausforderungen konfrontiert. Diese und weitere ethische Fragen wurden im Rahmen einer umfangreichen Studie in Großbritannien untersucht. Die Studienergebnisse wurden von der ICPHR in der Form von Leitsätzen veröffentlicht [35]. Darüber hinaus wurden aus den Reihen der ICPHR [36] ethische Fallstudien aus partizipativen Forschungsprojekten verschiedener Länder zusammengetragen, um kontextspezifische Auseinandersetzungen zu reflektieren. In Ländern, wo Forschungsprojekte routinemäßig um die Genehmigung einer Ethikkommission ersuchen müssen, stellen sich zudem Fragen der Zusammensetzung und der Arbeitsweise der Kommission: Hier wird die Beteiligung von Menschen außerhalb des Wissenschaftsbetriebs gefordert und/oder es werden eigenständige Ethikkommissionen außerhalb von Hochschulen (Community Review Boards) eingerichtet [37].

## Fazit

PGF etabliert sich als Ansatz in den Gesundheitswissenschaften, der Wissenschaft und Praxis auf besondere Weise verbindet. Dies geschieht durch die Einbeziehung von Menschen außerhalb wissenschaftlicher Einrichtungen als gleichberechtigte Forschungspartner*innen. Die PGF befindet sich international und auch in Deutschland in einer dynamischen Phase der Weiterentwicklung. Dabei werden Fragen der Definition, der Methodik, der Ethik und des Mehrwerts geklärt. Vorsicht ist jedoch geboten, wenn sich die PGF zu rasch im Sinne einer Popularisierung entwickelt, indem die Partizipation als Bedingung der Förderung festgelegt wird. Dies kann zu Scheinpartizipation führen und den (notwendigerweise langsamen) Aufbau von dauerhaften Partnerschaften zwischen verschiedenen Einrichtungen und Interessengruppen verhindern. Die zunehmende internationale Vernetzung von partizipativ Forschenden bietet zahlreiche Gelegenheiten, grundlegende Fragen der PGF zu beantworten und die Qualität ihrer Praxis zu fördern.

## Footnotes

1 Die Kritische Theorie ist eine gesellschaftskritische Richtung in den Sozial- und Geisteswissenschaften, deren Ursprünge vor allem in der Arbeit von Karl Marx, Georg Wilhelm Friederich Hegel und Sigmund Freud liegen. Der Begriff wurde in den 1930er-Jahren von der Frankfurter Schule geprägt, einer Gruppe von Wissenschaftlern, die am Institut für Sozialforschung in Frankfurt am Main ihren Sitz hatte und zu der u. a. Max Horkheimer, Theodor Adorno, Herbert Marcuse und Erich Fromm gehörten.
